# Standard filtration practices may significantly distort planktonic microbial diversity estimates

**DOI:** 10.3389/fmicb.2015.00547

**Published:** 2015-06-02

**Authors:** Cory C. Padilla, Sangita Ganesh, Shelby Gantt, Alex Huhman, Darren J. Parris, Neha Sarode, Frank J. Stewart

**Affiliations:** School of Biology, Georgia Institute of TechnologyAtlanta, GA, USA

**Keywords:** marine bacteria, particle-associated, Vibrio, richness, filter fractionation, prefilter

## Abstract

Fractionation of biomass by filtration is a standard method for sampling planktonic microbes. It is unclear how the taxonomic composition of filtered biomass changes depending on sample volume. Using seawater from a marine oxygen minimum zone, we quantified the 16S rRNA gene composition of biomass on a prefilter (1.6 μm pore-size) and a downstream 0.2 μm filter over sample volumes from 0.05 to 5 L. Significant community shifts occurred in both filter fractions, and were most dramatic in the prefilter community. Sequences matching Vibrionales decreased from ~40 to 60% of prefilter datasets at low volumes (0.05–0.5 L) to less than 5% at higher volumes, while groups such at the Chromatiales and Thiohalorhabdales followed opposite trends, increasing from minor representation to become the dominant taxa at higher volumes. Groups often associated with marine particles, including members of the Deltaproteobacteria, Planctomycetes, and Bacteroidetes, were among those showing the greatest increase with volume (4 to 27-fold). Taxon richness (97% similarity clusters) also varied significantly with volume, and in opposing directions depending on filter fraction, highlighting potential biases in community complexity estimates. These data raise concerns for studies using filter fractionation for quantitative comparisons of aquatic microbial diversity, for example between free-living and particle-associated communities.

## Introduction

Most studies of planktonic microbes begin by isolating organisms from the environment. This task usually involves collection of suspended biomass by passing water through a filter. For studies of planktonic bacteria and archaea, it is routine to use an inline series of filters during the collection step, often with a prefilter of larger pore size (typically 1.0–30 μm) upstream of a primary collection filter of smaller pore size (0.1–0.2 μm; Fuhrman et al., 1988; Venter et al., 2004; Walsh et al., 2009; Morris and Nunn, 2013; Stewart, 2013). Both filters retain microbial biomass, and the larger and smaller biomass size fractions are often used to approximate divisions between particle-associated and free-living microbes, respectively (e.g., Acinas et al., 1999; Crump et al., 1999; Hollibaugh et al., 2000; Moeseneder et al., 2001; LaMontagne and Holden, 2003; Ghiglione et al., 2007, 2009; Rusch et al., 2007; Kellogg and Deming, 2009; Smith et al., 2013; D'Ambrosio et al., 2014; Ganesh et al., 2014; Mohit et al., 2014; Orsi et al., 2015, among others). Retained biomass can be used toward a range of analytical goals, including quantitative measurements of community taxonomic and metabolic diversity, based for example on 16S rRNA gene or metagenome sequencing. Such analyses shape our understanding of microbial distributions and functions in the environment.

The effects of filtration method on quantitative measurements of community genetic diversity are poorly understood, and rarely accounted for in study design. Notably, it is unclear how prefiltration and variation in filtered water volume influence the relative abundances of taxa retained in different filter fractions. This is surprising given an extensive literature demonstrating variation in particle retention and clogging rates among filter types and in the size spectra of retained particles due to clogging (Nagata, 1986; Taguchi and Laws, 1988; Knefelkamp et al., 2007), and a smaller number of studies showing differential selection of target taxa based on filter type (Gasol and Moran, 1999), or variation in DNA extraction efficiency depending on filtered water volume (Boström et al., 2004). Indeed, sample volume varies widely across studies, from less than 1 L for coastal or eutrophic environments (Hollibaugh et al., 2000) to greater than 100 L for open ocean settings (Rusch et al., 2007). Fewer studies sample at very low volumes (microliters), in part to avoid under-sampling community richness, potentially due to microscale heterogeneity in biomass distributions (Long and Azam, 2001). Sample volume is often also variable among samples within a study, differing for instance between samples collected for DNA vs. RNA analysis (Frias-Lopez et al., 2008; Hunt et al., 2013). While it has been assumed that the proportion of free-living bacteria retained in the prefilter fraction increases with the volume of filtered water (Lee et al., 1995), the effects of such retention on community composition measurements are unknown. Advances in high-throughput 16S rRNA gene sequencing now enable rapid, low-cost quantification of microbial community structure (Caporaso et al., 2011). These methods can be used to help identify biases associated with common sample collection practices.

We conducted experiments to test whether variation in the volume of filtered seawater biases estimates of marine microbial diversity. Biomass representing two size fractions (0.2–1.6 μm, >1.6 μm) was collected from a marine oxygen minimum zone (OMZ) following a sequential inline filtration protocol used routinely to study planktonic microbes (Fuhrman et al., 1988; Frias-Lopez et al., 2008; Stewart, 2013). Sequencing of community 16S rRNA gene amplicons revealed significant shifts in community structure and richness over volumes ranging from 0.05 to 5 L. We discuss these results as evidence that sample volume should be critically examined as a potentially confounding variable in comparing estimates of aquatic microbial diversity.

## Materials and methods

### Sample collection and filtration

Seawater was collected from the OMZ off Manzanillo, Mexico during the Oxygen Minimum Zone Microbial Biogeochemistry Expedition 2 (OMZoMBiE2) cruise (*R/V New Horizon*; cruise NH1410; May 10-June 8, 2014). Collections were made using 30 L Niskin bottles attached to a CTD-Rosette. Seawater was emptied into 20 L cubitainers upon retrieval on deck and stored in the dark at 4°C until filtration (<1 h).

Two experiments were conducted to assess the impact of sample volume on microbial diversity. Experiment 1 collected biomass after filtration of 0.1, 1, and 5 L from the sample pool, with 3–5 replicate filtration lines per volume. Experiment 2 examined volumes of 0.05, 0.1, 0.5, 1, 2, and 5 L, with 2–3 replicates per volume. These ranges encompass volumes typical for studies of both coastal and open ocean environments, although fewer studies collect volumes at the lower end of the range. Water for experiment 1 was collected from depth 150 m near the secondary nitrite maximum at a site ~95 km offshore from Manzanillo (18′12″N 104′12″W). Water for experiment 2 was collected from depth 400 m at a site ~300 km offshore from Manzanillo (20′00″ N, 107′00″ W). Collections depths at both sites were beneath the photic zone. Total water depth at both sites was >1000 m.

For each experiment, discrete water volumes were measured from the stored sample water, transferred into pre-washed secondary containers (graduated cylinders or glass bottles), and filtered by sequential in-line filtration at room temperature through a glass fiber disc pre-filter (GF/A, 47 mm, 1.6 μm pore-size, Whatman) and a primary collection filter (Sterivex-GP filter cartridge, polyethersulfone membrane, 0.22 μm pore-size, Millipore) using a peristaltic pump (Masterflex L/S modular drive pump, 1–100 rpm, Cole-Parmer®) at constant speed (60 rpm). Replicate filters were collected in parallel (separate filter lines) for each discrete volume, with a maximum of 5 lines (replicates) running in parallel at once. After filtration, prefilters were transferred to cryovials containing lysis buffer (~1.8 ml; 50 mM Tris-HCl, 40 mM EDTA, and 0.73 M Sucrose), Sterivex cartridges were filled with lysis buffer (~1.8 ml) and capped at both ends, and both filter types were stored at −80°C until DNA extraction. After filtration of one set of replicates, filter lines were rinsed, and clean filters were added. Aliquots representing the next sample in the volume series were then measured from the source water, and the filtration sequence was repeated.

### DNA extraction

DNA was extracted from prefilters and Sterivex cartridges using a phenol:chloroform protocol as in Ganesh et al. (2014). Cells were lysed by adding lysozyme (2 mg in 40 μl of lysis buffer per filter) directly to the prefilter-containing cryovials and the Sterivex cartridges, sealing the caps/ends, and incubating for 45 min at 37°C. Proteinase K (1 mg in 100 μl lysis buffer, with 100 μl 20% SDS) was added, and cryovials and cartridges were resealed and incubated for 2 h at 55°C. The lysate was removed, and DNA was extracted once with Phenol:Chloroform:Isoamyl Alcohol (25:24:1) and once with Chloroform:Isoamyl Alcohol (24:1) and then concentrated by spin dialysis using Ultra-4 (100 kDa, Amicon) centrifugal filters. Double stranded DNA was quantified on a Qubit® Fluorometer using the dsDNA BR Assay Kit.

### 16S rRNA gene quantitative PCR

Quantitative PCR (qPCR) was used to count total bacteria 16S rRNA gene copies in DNA extracted from both prefilter and Sterivex size fractions. Total 16S counts were obtained using SYBR® Green-based qPCR and universal bacterial 16S primers 1055f and 1392r, as in Hatt et al. (2013). Ten-fold serial dilutions of DNA from a plasmid carrying a single copy of the 16S rRNA gene (from *Dehalococcoides mccartyi*) were included on each qPCR plate and used to generate standard curves. Assays were run on a 7500 Fast PCR System and a StepOnePlus™ Real-Time PCR System (Applied Biosystems). All samples were run in triplicate (20 μL each) and included 1X SYBR® Green Supermix (BIO-RAD), 300 nM of primers, and 2 μL of template DNA (diluted 1:100). Thermal cycling involved: incubation at 50°C for 2 min to activate uracil-N-glycosylase, followed by 95°C for 10 min to inactivate UNG, denature template DNA, and activate the polymerase, followed by 40 cycles of denaturation at 95°C (15 s) and annealing at 60°C (1 min).

### Diversity analyses

High-throughput sequencing of dual-indexed PCR amplicons encompassing the V4 region of the 16S rRNA gene was used to assess microbial community composition. Briefly, amplicons were synthesized using Platinum® PCR SuperMix (Life Technologies) with primers F515 and R806, encompassing the V4 region of the 16S rRNA gene (Caporaso et al., 2011). These primers are used primarily for bacterial 16S rRNA genes analysis, but also amplify archaeal sequences. Both forward and reverse primers were barcoded and appended with Illumina-specific adapters according to Kozich et al. (2013). Equal amounts of starting DNA (0.5 ng) were used for each PCR reaction to avoid potential PCR biases due to variable template concentrations (Kennedy et al., 2014). Thermal cycling involved: denaturation at 94°C (3 min), followed by 30 cycles of denaturation at 94°C (45 s), primer annealing at 55°C (45 s) and primer extension at 72°C (90 s), followed by extension at 72°C for 10 min. Amplicons were analyzed by gel electrophoresis to verify size (~400 bp, including barcodes and adaptor sequences) and purified using the Diffinity RapidTip2 PCR purification tips (Diffinity Genomics, NY). Amplicons from different samples were pooled at equimolar concentrations and sequenced on an Illumina MiSeq using a 500 cycle kit with 5% PhiX to increase read diversity.

Amplicons were analyzed using QIIME (Caporaso et al., 2010) following standard protocols. Barcoded sequences were de-multiplexed and trimmed (length cutoff 100 bp) and filtered to remove low quality reads (average Phred score <25) using Trim Galore! (http://www.bioinformatics.babraham.ac.uk/projects/trim_galore/). Paired-end reads were then merged using FLASH (Magoč and Salzberg, 2011), with criteria of average read length 250, fragment length 300, and fragment standard deviation 30. Chimeric sequences were detected by reference-based searches using USEARCH (Edgar, 2010). Identified chimeras were filtered from the input dataset, and merged non-chimeric sequences were clustered into Operational Taxonomic Units (OTUs) at 97% sequence similarity using open-reference picking with the UCLUST algorithm (Edgar, 2010) in QIIME. The number of sequences assigned to OTUs averaged 22,693 (range: 2255–182,051) and 20,638 (range: 5152–46,634) per sample for experiments 1 and 2, respectively.

Taxonomy was assigned to representative OTUs from each cluster using the Greengenes database (DeSantis et al., 2006). OTU counts were rarefied (10 iterations) and alpha diversity was quantified at a uniform sequence depth (*n* = 2255 for exp 1 and 5152 for exp 2) using the Chao1 estimator of richness. Significant differences in Chao1 estimates between volume groupings were detected using pairwise two-sample *t*-tests with a Bonferroni correction for multiple comparisons. To compare community composition between samples, sequences were aligned in QIIME using PyNAST, and beta diversity was calculated using the weighted Unifrac metric (Lozupone and Knight, 2005). Sample relatedness based on Unifrac was visualized with two-dimensional Principal Coordinate Analyses. The statistical significance of sample grouping according to filtered volume was assessed using the nonparametric adonis method in QIIME based on Unifrac distances run with 999 permutations and Bonferroni corrections for multiple tests (Table S1).

Taxa (Order level) differing significantly in proportional abundance in pairwise comparisons of filter volumes (lowest vs. highest volumes) were identified via an empirical Bayesian approach using the program baySeq (Hardcastle and Kelly, 2010), as in Stewart et al. (2012) and Ganesh et al. (2014). The baySeq method assumes a negative binomial distribution with prior distributions derived empirically from the data (100,000 iterations). Dispersion was estimated via a quasi-likelihood method, with the count data normalized by dataset size (total number of identifiable 16S rRNA gene amplicons per dataset). Posterior likelihoods per Order were calculated for models (volume groupings) in which Orders were either predicted to be equivalently abundant between lowest vs. highest filter volumes or differentially abundant. A false discovery rate threshold of 0.05 was used for detecting differentially abundant categories.

All sequence data have been submitted to the Sequence Read Archive at NCBI under BioProject ID PRJNA275901.

## Results

**Figure 1:**
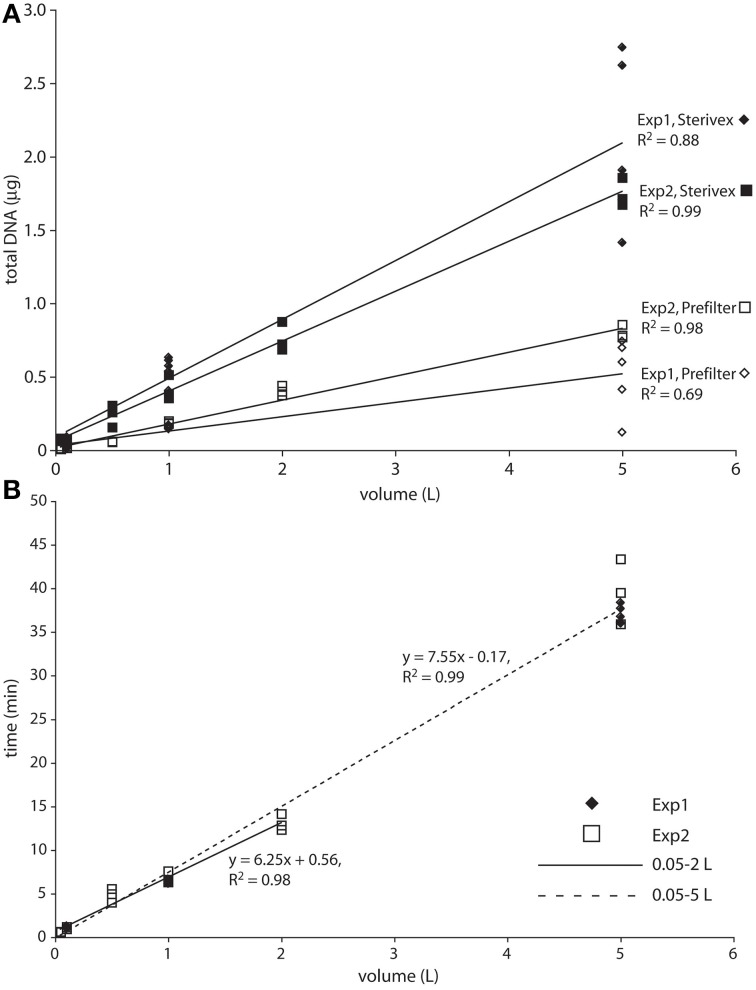
**Total DNA yield (A) and filtration time (B) as a function of filtered water volume**. The solid regression line in *B* spans the 0.05–2 L range. The dashed regression spans the full dataset, with the increased slope suggesting a slight decrease in flow rate at the highest volumes.

Total bacterial 16S rRNA gene counts (prefilter + Sterivex combined), a rough proxy for microbial abundance, averaged 4 × 10^5^ and 7 × 10^4^ (averaged across all volumes and replicates) for experiment 1 and 2, respectively, and were 10 to 11-fold higher in the Sterivex size fraction (0.2–1.6 μm; Table S1). These counts are within the range of or slightly lower than prior bacterial 16S gene counts at OMZ and oxycline depths in the study area (~5 × 10^4^ to 1.3 × 10^6^; Ganesh et al., 2015; Stewart, unpublished), values typical of open ocean waters, and consistent with prior reports of elevated microbial biomass in the smallest (“free-living”) size fraction compared to particulate size fractions (Cho and Azam, 1988; Ghiglione et al., 2009). DNA yields, representing both prokaryote and eukaryote sources, were ~2 to 4-fold higher in Sterivex filter fractions compared to prefilters (Figure 1A). Both DNA yields and bacterial 16S counts increased linearly with volume in both fractions, and were most variable (among replicates) for the 5 L samples, suggesting differences in extraction efficiency at high volumes (Figure 1A, Figure S1). Similarly, filtration time increased linearly as a function of sample volume (*R*^2^ = 0.99; Figure 1B), ranging from <40 s (0.05 L) to 43 min (5 L). Average flow rate, calculated from filtration times from all samples, was 0.12 L min^−1^. A minor increase of filtration times per unit volume was observed for the 5 L samples compared to 1 and 2 L samples, highlighting a very slight decline in flow rate with volume, potentially due to filter clogging. No filters visibly ruptured during the experiments.

**Figure 2:**
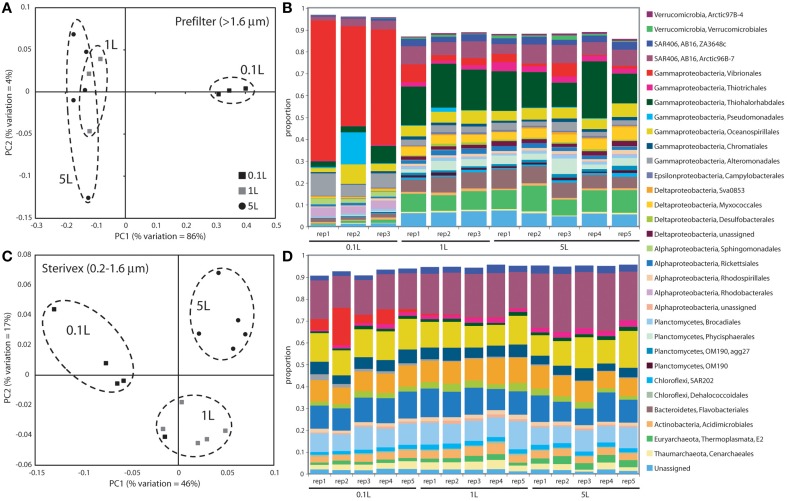
**Microbial community relatedness (A,C) and taxon abundances (B,D) in experiment 1 prefilter (>1.6 μm; A,B) and Sterivex (0.2–1.6 μm; C,D) samples**. Relatedness based on 16S rRNA gene amplicon sequencing, as quantified by the weighted Unifrac metric. Samples representing different filtered water volumes are circled. Abundances in **(B,D)** are percentage abundances of major bacterial Orders. Only Orders with abundance >0.5% (averaged across all replicates) are shown.

**Figure 3:**
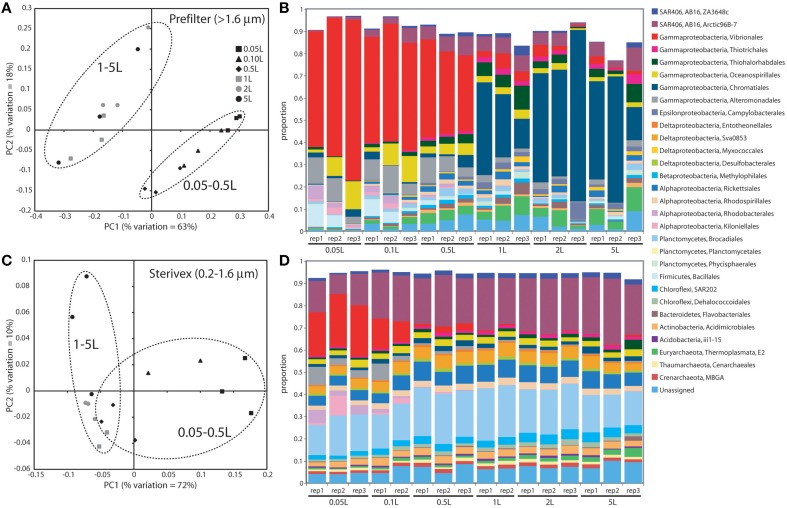
**Microbial community relatedness (A,C) and taxon abundances (B,D) in experiment 2 prefilter (>1.6 μm; A,B) and Sterivex (0.2–1.6 μm; C,D) samples**. Relatedness based on 16S rRNA gene amplicon sequencing, as quantified by the weighted Unifrac metric. Samples representing different filtered water volumes are circled. Abundances in **(B,D)** are percentage abundances of major bacterial Orders. Only Orders with abundance >0.5% (averaged across all replicates) are shown.

**Figure 4:**
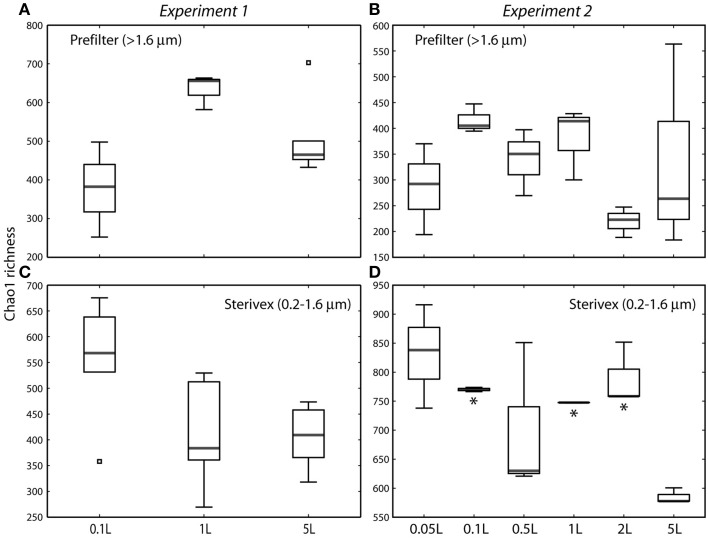
**Chao1 estimates of operational taxonomic unit (97% similarity cluster) richness in prefilter (>1.6 μm; A,B) and Sterivex (0.2–1.6 μm; C,D) samples in experiments 1 and 2**. Boxplots show medians within first and third quartiles, with whiskers indicating the lowest and highest values within 1.5 times the interquartile range of the lower and upper quartiles, respectively. Note variation in y-axis scales. Asterisks in **(D)** indicate volume groupings with richness estimates significantly different from those of the 5 L sample (*P* < 0.05). All other pairwise differences between volume groupings were not statistically significant.

Community structure varied significantly with sample volume in both biomass fractions. Principal coordinates analysis based on the weighted Unifrac metric showed significant differentiation of samples based on filtered volume for both size fractions in both experiments (*R*^2^: 0.50–0.87; *P* < 0.05), with the strongest separation occurring between samples of 1 L or more and those of lower volumes (Figures 2, 3; Table S2). Chao1 estimates of operational taxonomic unit (OTU; 97% similarity cluster) richness varied with filter volume in both size fractions (Figure 4). In both experiments, median richness in the >1.6 μm fraction increased (by up to 68%) with volume, reaching peak values at 1 L, before declining again at the highest volumes (2–5 L). However, differences in prefilter richness between volume groupings were not significant, due partly to limited replication per volume. In contrast, in both experiments richness in the downstream 0.2–1.6 μm “Sterivex” size fraction declined by ~25–30% over the volume range, with statistically significant declines observed between lower volumes and the 5 L samples in experiment 2 (*P* < 0.05).

The most dramatic shifts in taxonomic representation with volume occurred in the prefilter fraction (Figures 2, 3). Of the major Prokaryotic Orders detected in experiment 1, where “major” here indicates an average percent abundance >0.5% across filter type replicates, 59% (19 of 32) varied significantly in abundance from lowest to highest volumes (0.1 vs. 5 L; *P* < 0.05), with 88% (28) undergoing a fold-change of 2 or greater (positive or negative; Table S3). In experiment 2, 96% of major Orders underwent a fold-change of 2 or greater over the volume range (0.05 vs. 5 L), although fewer (17%) of these changes were statistically significant (likely due to fewer replicates per volume in experiment 2; Table S4). In both experiments, sequences matching Vibrionales decreased dramatically (21–33 fold) with filter volume, from >50% (average) of the lowest volume datasets, to <3% at 5 L. In contrast, other groups increased to dominate the dataset over the volume range. In experiment 1, sequences related to the gammaproteobacterial Thiohalorhabdales increased nearly 4-fold, becoming the single most abundant Order at 5 L with 17% of total sequences. In experiment 2, the Order Chromatiales increased from an average of 2% at lower volumes (range: 0.2–5.7% over 0.05, 0.1, 0.5 L datasets) to become the most abundant taxon at higher volumes, representing an average of 42% of total sequences at volumes of 1 L or greater (range: 15–77%; Figure 3, Table S4). In both experiments, Euryarchaeota of the Thermoplasmata increased 10 to 20-fold to become the second most abundant Order (7–10%) at 5 L. Diverse members of the Deltaproteobacteria, Planctomycetes, and Bacteroidetes also underwent substantial increases (4 to 27-fold) in the prefilter fraction in both experiments (Tables S3, S4).

Large changes in taxon representation also occurred in the Sterivex fraction, but were less dramatic that those in the prefilter community. In both experiments, 14% of the major prokaryotic Orders detected in this fraction changed significantly in representation over the volume range (*P* < 0.05, Tables S3, S4). As in the prefilter datasets, the most substantial shifts occurred in the Vibrionales, which decreased 51 to 97-fold with volume. Other groups undergoing significant declines included the Alteromonadales, Rhodobacterales, and Kiloniellales, notably with these groups declining 16 to 94-fold in experiment 2. Less dramatic, but still substantial, shifts occurred in the opposite direction, notably with the abundant SAR406 lineage increasing ~2-fold in representation in both experiments to constitute over one quarter of all sequences in the 5 L datasets.

## Discussion

Studies of diverse aquatic habitats consistently show that microbial community composition in the prefilter fraction differs from that of the smaller size fraction (e.g., Simon et al., 2002, 2014, and references therein). These differences have been used to suggest taxonomic, chemical, and metabolic partitioning between particle-associated vs. free-living microbial communities (see references in Introduction). While the pore size of the primary collection filter used in such studies is almost always 0.2 μm, the pore size of the prefilter varies, typically from 0.8 to 1.0 (e.g., Hollibaugh et al., 2000; Allen et al., 2012) to 30 μm (e.g., Fuchsman et al., 2011, 2012), but is most often in the range of 1 to 3 μm, encompassing the 1.6 μm GF/A prefilter used in this study. Here, sample volume had a much stronger effect on prefilter community composition compared to that of the downstream Sterivex fraction. Taxa such as the Thiohalorhabdales and Chromatiales in experiments 1 and 2, respectively, were evenly distributed between filter fractions at low volumes but dramatically enriched in the prefilter at higher volumes. Furthermore, major taxonomic divisions often described as being enriched in particulate fractions, including the Flavobacteriales (Bacteroidetes), Myxococcales (Deltaproteobacteria), and Planctomycetes (Crump et al., 1999; Simon et al., 2002; Eloe et al., 2011; Allen et al., 2012; Fuchsman et al., 2012), were among those whose proportional abundances in the prefilter fraction increased the most with filter volume, although these groups have also been detected on particles sampled directly by syringe (DeLong et al., 1993; Rath et al., 1998). Such changes were not exclusive to the prefilter fraction. SAR406, an uncultured lineage commonly affiliated with low-oxygen waters (Wright et al., 2012, 2014), increased steadily with volume to become the single most abundant taxonomic group in the Sterivex fraction in both experiments. These patterns suggest that differences in sample volume can bias multiple sequential filter fractions, but may be particularly problematic for studies inferring the structure of the particle-associated community, or the association of certain taxa with a particle-attached niche.

Community diversity differences between particle-associated and free-living biomass fractions are often variable across diverse ocean habitats. This has been attributed to environmental variation associated with geographic and vertical zonation, as well as flexibility in microbial lifestyles allowing alternation between particle-associated and free-living lifestyles (Grossart, 2010). Surprisingly, only a small number of studies have evaluated the potential for collection methods to bias community-level diversity measurements (Kirchman et al., 2001; Long and Azam, 2001; Ghiglione et al., 2007). Focusing on the 0.2–3.0 μm filter fraction, Long and Azam (2001) recovered qualitatively similar community 16S rRNA gene fingerprints (denaturing gradient gel electrophoresis; DGGE) from coastal seawater volumes ranging from 1 μl to 1 L. Similarly, Ghiglione et al. (2007) reported no visible effect of volume (0.1 to 5 L) on fingerprints (capillary electrophoresis-single strand conformation polymorphism) from Mediterranean Sea bacterioplankton on 0.2 and 0.8 μm filters. High throughout 16S amplicon sequencing now provides a cost-competitive alternative to fingerprinting with higher sensitivity for detecting community structural shifts, including those involving rare taxa. Using this method, our results suggest that biases due to variation in filtered volume may also confound comparisons of size-fractionated community structure across habitats and studies.

Our data also suggest that metrics of community (OTU) richness may be sensitive to filtered water volume. In both experiments, richness of the prefilter and Sterivex communities followed opposing trends with volume, increasing in the larger size fraction and decreasing in the smaller. Declines in diversity with filter volume (0.01 vs. 2 L) were previously observed in a study of prefiltered bacterioplankton (0.2–3.0 μm fraction) using DGGE, although determinants of that decline could not be related directly to volume due to variations in the DNA extraction procedure (Kirchman et al., 2001). Here, in experiment 1, richness of the prefilter fraction was lower than that of the Sterivex fraction at 0.1 L, but this pattern was reversed at higher volumes. Enhanced richness in prefilter vs. 0.2 μm fractions has been reported from diverse systems (Eloe et al., 2011; Bižić-Ionescu et al., 2014; Ganesh et al., 2014), leading to speculation that the complexity of the microbial community varies differentially between free-living and particle-associated niches in response to fluctuation in water column conditions or niche-availability on particles. Bulk estimates of community complexity may instead be driven by variation in filtered water volume.

The observed compositional shifts with sample volume are potentially driven by a range of mechanisms. First, the filters may be clogging. A minor increase in filtration time per unit sample volume for the 5 L samples compared to the lower volume samples is consistent with filter clogging, although this trend is not supported by the near linear increase in total DNA yield and bacterial 16S gene counts per volume (Figure 1A, Figure S1). If the filters are indeed clogging, clogging would presumably lead to a progressive increase in the size range and diversity of the retained particles, with smaller (potentially free-living) cells below the size threshold of the unclogged filter increasingly retained as volumes increase. We speculate, for example, that Chromatiales and Thiohalorhabdales cells or cell aggregates are potentially either near the size threshold for passage through the prefilter, or are otherwise easily entrained in the filter matrix, and are therefore preferentially retained if the prefilter clogs.

Clogging could also affect diversity estimates for the downstream Sterivex filter fraction. In this fraction, however, effects due to clogging and increased retention of cells <0.2 μm are presumably minimal, as few planktonic marine microbes likely pass through the 0.2 μm filter to begin with (Fuhrman et al., 1988), although the abundance of very small cells in the open ocean remains an active area of research. Rather, the community shifts in the Sterivex fraction could be driven by clogging of the upstream prefilter, with the decline in OTU richness with filter volume observed in both experiments potentially reflecting a selective narrowing of the range of taxa making it through the prefilter. Taxa that increase in abundance with volume in the 0.2 μm fraction, such as SAR406, may be relatively small cells, which presumably would be increasingly selected for as the prefilter clogs.

Second, filtration of small volumes, by chance, may fail to capture a representative subset of the particulate fraction, due to microscale patchiness of bacterioplankton and particle distributions (Azam and Hodson, 1981; Azam and Ammerman, 1984; Mitchell and Fuhrman, 1989). Marine particles span gradients of age, size, and source material, and potentially microbial composition (Simon et al., 2002). It is possible that certain particles, due to their low abundance, are unlikely to be captured at low water volumes, but would be sampled at higher volumes, and potentially contribute significantly to bulk microbial counts depending on the local density and richness of microbes on the particle. Changes in taxon representation due to increases in water volume may not be easily distinguished from changes due to filter clogging.

Third, adsorption of free dissolved DNA to filters may contribute disproportionately to diversity estimates at smaller volumes (Turk et al., 1992; Boström et al., 2004). While we cannot rule out this possibility, proportional decreases of filter-bound DNA with volume seems an unlikely explanation for the observed taxonomic shifts, as the dissolved DNA pool would need to be dominated almost exclusively by Vibrionales for this to be true. Also, DNA extraction efficiency has been shown to decrease with filtered water volume (Boström et al., 2004), suggesting that variable extraction efficiencies may affect community composition estimates. A consistent decrease in extraction efficiency with volume was not clearly evident in our samples, although the range in DNA recovered from 5 L samples indicates that efficiency was variable (Figure 1A). Although the potential for extraction efficiency to change with sample volume deserves more attention, and is likely variable among filter types and extraction methods, decreasing efficiencies would only alter composition estimates if the change in efficiency is non-uniform among taxa. It is unknown if this is true.

Finally, it is possible that population turnover (growth or lysis) in the source water during filtering may also have contributed to compositional shifts with volume. We consider this possibility to be unlikely. Growth in the source water was likely negligible given characteristic doubling times of marine microbes (hours to days; Ducklow, 2000) and the low temperature of the stored source water (stored at 4°C, but filtered at room temperature). In both experiments, the total processing time of 0.1 and 1 L samples was less than 15 min. Growth by even the most active microbes under optimal conditions would be unlikely to result in the dramatic community structural shifts observed between these sample volumes (Figures 2, 3). Furthermore, the observed taxonomic shifts are inconsistent with changes due to differential turnover of microbial groups. Taxa typically associated with rapid growth during bottle incubations and in response to organic matter enrichment, notably members of the Alteromonadales, Pseudomonadales, and Vibrionales (Pinhassi and Berman, 2003; Allers et al., 2007, 2008; Stewart et al., 2012), decreased in abundance with increasing volume in both experiments (Tables S3, S4), inconsistent with results due to rapid growth during filtering. Furthermore, had the observed changes been due to growth, or to differential lysis of cells during sampling, we might predict changes of similar magnitude in both biomass fractions. This was not the case, as the most dramatic changes were observed in the prefilter biomass, consistent with effects due to filter clogging.

## Conclusion

These results, based on biomass from an open ocean environment and collected following a widely used filtration scheme, highlight a potential for sample volume variation to confound community diversity estimates. However, it is unlikely that our results extrapolate evenly to all systems. The magnitude and exact mechanism of sample volume-based biases likely differ depending on filter type and pore size, pumping rate, community composition, bulk microbial abundance, particle load, and potentially other variables. Such biases may be even greater in waters more eutrophic than those tested here, and may not easily be eliminated by frequent replacement of prefilters. For studies comparing particle-associated vs. free-living communities, elimination of the prefiltration step may be necessary, with collection of particle communities based instead on direct sampling of particles without filtration (DeLong et al., 1993), potentially via gravity or flow-cytometric separation. Such alternative methods should be explored. Our data suggest that measurements of community structure for biomass separated by filter fractionation should only be considered accurate and used for quantitative comparisons when effects of sample volume variation are shown to be negligible.

### Conflict of interest statement

The authors declare that the research was conducted in the absence of any commercial or financial relationships that could be construed as a potential conflict of interest.
