# Supplementary material for: Standard filtration practices may significantly distort planktonic microbial diversity estimates
Source: Front Microbiol. 2015 Jun 2;6:547. doi: 10.3389/fmicb.2015.00547 (PMC4451414; doi:10.3389/fmicb.2015.00547)
Supplement: Supplementary file 1 [file Supplementary_Material.pdf]

Supplementary Material:

Standard filtration practices may significantly distort planktonic microbial diversity estimates

Cory Padilla, Sangita Ganesh, Shelby Gantt, Alex Huhman, D. Joshua Parris, Neha Sarode, Frank J. Stewart

Table S1. Bacterial 16S rRNA gene copies per mL in sample water from experiments 1 and 2. Values are averages across all volume replicates, with standard deviation in parentheses. The ratio of Sterivex to prefilter counts is shown in the last column.

exp	Sterivex (0.2-1.6 μm)	prefilter (>1.6 μm)	combined	ratio (Sterivex/prefilter)
1-150m	3.7×10^5 (1.1×10^5)	3.3×10^4 (1.4×10^4)	4.0×10^5 (1.2×10^5)	11.1
2-400m	6.4×10^4 (2.2×10^4)	6.3×10^3 (1.4×10^3)	7.0×10^4 (2.4×10^4)	10.3

Figure S1. Total bacterial 16S rRNA gene counts as a function of filtered water volume.

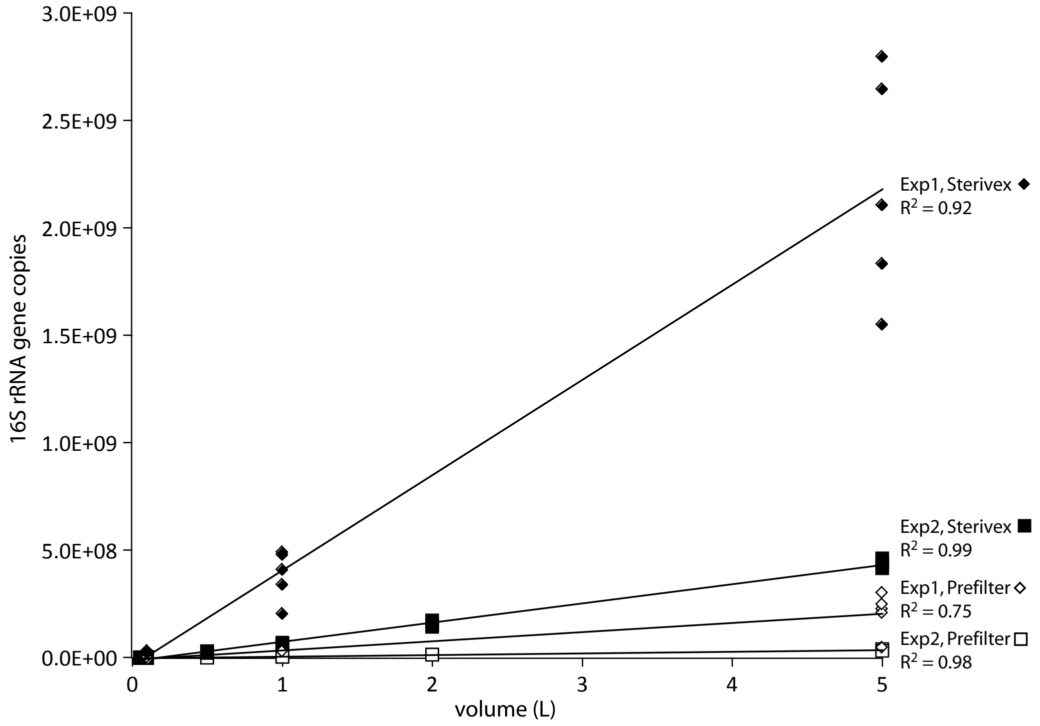

Table S2. Percentage variation (R^2) in weighted UniFrac distances explained by filtered water volume differences, based on adonis tests in QIIME. All P-values are significant following Bonferroni correction for multiple tests.

dataset	R^2, *P-value*
Exp1, prefilter	0.87, *0.01*
Exp1, Sterivex	0.50, *0.001*
Exp2, prefilter	0.74, *0.001*
Exp2, Sterivex	0.81, *0.001*

Table S3. Abundance of microbial orders in ***experiment 1***, expressed as a % of total 16S rRNA gene amplicons.

* average % of replicates
**positive values: fold increase in abundance from the lowest to the highest filtered volumes;
**negative values: fold increase from the highest to the lowest filtered volumes, multiplied by -1
Shading = significant difference in abundance between lowest vs highest volumes ($P<0.05$; FDR; baySeq)

Sterivex filter fraction (0.2-1.6 μm)

	Filtered water volume*				
Taxon	0.1L	1L	5L	Average (all)	Fold Change**
SAR406, Arctic96B-7	16.2	18.8	25.1	20.0	1.6
Gammaproteobacteria, Oceanospirillales	12.9	11.2	12.5	12.2	-1.0
Alphaproteobacteria, Rickettsiales	11.2	11.5	10.6	11.1	-1.1
Deltaproteobacteria, Sva0853	9.0	10.6	9.1	9.6	1.0
Planctomycetes, Brocadiales	9.0	10.0	8.5	9.2	-1.1
Gammaproteobacteria, Chromatiales	5.3	5.4	4.3	5.0	-1.2
Actinobacteria, Acidimicrobiales	3.0	3.9	3.2	3.4	1.0
SAR406, AB16, ZA3648c	2.1	3.0	3.3	2.8	1.6
Thaumarchaeota, Cenarchaeales	2.7	3.4	2.2	2.8	-1.2
Deltaproteobacteria, Desulfobacterales	2.3	3.2	2.4	2.6	1.0
Gammaproteobacteria, Vibrionales	6.7	0.3	0.1	2.4	-96.5
Euryarchaeota, Thermoplasmata, E2	1.5	2.0	3.2	2.2	2.2
Unassigned	2.1	2.0	1.8	2.0	-1.2
Gammaproteobacteria, Thiotrichales	1.5	1.9	2.5	1.9	1.7
Chloroflexi, SAR202	1.6	2.1	2.0	1.9	1.3
Alphaproteobacteria, Rhodospirillales	1.4	1.8	1.5	1.6	1.0
Gammaproteobacteria, Thiohalorhabdales	1.1	1.4	1.6	1.4	1.4
Alphaproteobacteria, unassigned	0.8	1.2	0.8	0.9	-1.0
Gammaproteobacteria, Alteromonadales	1.5	0.6	0.4	0.8	-3.6
Chloroflexi, Dehalococcoidales	0.6	0.7	0.5	0.6	-1.1
Bacteroidetes, Flavobacteriales	0.4	0.4	0.6	0.5	1.5

Table S3 continued

Prefilter fraction (>1.6 μm)

	Filtered water volume*				
Taxon	0.1L	1L	5L	Average (all)	Fold Change
Gammaproteobacteria, Vibrionales	54.1	4.6	2.6	17.2	-20.5
Gammaproteobacteria, Thiohalorhabdales	4.4	18.8	17.0	14.1	3.8
Euryarchaeota, Thermoplasmata, E2	1.0	7.9	9.6	6.8	9.8
SAR406, Arctic96B-7	3.1	7.3	6.9	6.0	2.2
Bacteroidetes, Flavobacteriales	1.5	6.7	7.0	5.4	4.7
Gammaproteobacteria, Oceanospirillales	4.7	5.2	5.1	5.0	1.1
Unassigned	1.3	6.7	6.1	4.9	4.6
Gammaproteobacteria, Alteromonadales	7.4	2.8	2.6	4.0	-2.8
Deltaproteobacteria, Myxococcales	0.5	3.6	4.2	3.1	8.4
Planctomycetes, Phycisphaerales	0.2	3.0	3.9	2.7	15.9
SAR406, ZA3648c	0.7	3.1	3.0	2.4	4.1
Gammaproteobacteria, Thiotrichales	0.3	1.8	2.9	1.9	8.7
Gammaproteobacteria, Pseudomonadales	5.2	0.8	0.2	1.7	-21.6
Alphaproteobacteria, Rickettsiales	0.9	2.1	1.9	1.7	2.0
Deltaproteobacteria, unassigned	0.1	1.6	2.0	1.4	27.4
Alphaproteobacteria, Rhodobacterales	3.9	0.2	0.3	1.2	-12.9
Gammaproteobacteria, Chromatiales	0.6	1.3	1.3	1.1	2.0
Planctomycetes, Brocadiales	0.7	1.2	1.2	1.1	1.6
Alphaproteobacteria, Rhodospirillales	0.6	1.1	1.3	1.1	2.2
Planctomycetes, OM190, agg27	0.1	1.0	1.4	0.9	15.5
Actinobacteria, Acidimicrobiales	0.7	1.1	0.8	0.9	1.2
Chloroflexi, SAR202	0.3	0.9	1.0	0.8	3.6
Planctomycetes, OM190,	0.2	1.1	0.9	0.8	4.9
Verrucomicrobia, Verrucomicrobiales	0.1	0.6	1.1	0.7	7.9
Deltaproteobacteria, Desulfobacterales	0.4	1.0	0.7	0.7	2.0
Alphaproteobacteria, Sphingomonadales	2.1	0.2	0.1	0.7	-30.4
Thaumarchaeota, Cenarchaeales	0.3	0.7	0.7	0.6	2.6
Epsilonproteobacteria, Campylobacterales	0.2	0.7	0.7	0.6	3.1
Verrucomicrobia, Arctic97B-4	0.2	0.5	0.7	0.5	3.6
Deltaproteobacteria, Sva0853	0.4	0.4	0.7	0.5	1.8
Deltaproteobacteria, PB19	0.1	0.8	0.5	0.5	6.9
Gammaproteobacteria, Legionellales	0.2	0.6	0.5	0.5	2.8

Table S4. Abundance of microbial orders in ***experiment 2***, expressed as a % of total 16S rRNA gene amplicons.

* average % of replicates
**positive values: fold increase in abundance from the lowest to the highest filtered volumes;
**negative values: fold increase from the highest to the lowest filtered volumes, multiplied by -1
Shading = significant difference in abundance between lowest vs highest volumes (P<0.05; FDR; baySeq)

Sterivex filter fraction (0.2-1.6 μm)

	Filtered water volume*							
Taxon	0.05L	0.1L	0.5L	1L	2L	5L	Average (all)	Fold Change**
SAR406, Arctic96B-7	12.3	14.4	21.2	19.7	24.2	26.0	19.7	2.1
Planctomycetes, Brocadiales	16.0	11.3	21.7	19.7	20.0	15.6	17.4	-1.0
Unassigned	4.4	4.6	6.9	6.5	7.4	8.8	6.4	2.0
Alphaproteobacteria, Rickettsiales	4.5	3.9	7.6	7.0	7.5	6.7	6.2	1.5
Gammaproteobacteria, Vibrionales	22.0	7.8	2.4	0.6	0.2	0.4	5.6	-51.0
Gammaproteobacteria, Chromatiales	1.3	7.0	2.3	12.3	1.4	2.3	4.4	1.8
Deltaproteobacteria, Sva0853	3.6	3.1	5.4	4.9	5.1	4.3	4.4	1.2
Chloroflexi, SAR202	2.0	1.9	3.2	3.0	4.4	4.2	3.1	2.1
Actinobacteria, Acidimicrobiales	2.4	2.4	3.2	2.8	3.2	3.0	2.8	1.2
Gammaproteobacteria, Oceanospirillales	2.6	2.4	2.7	2.4	2.6	2.7	2.6	1.0
Alphaproteobacteria, Rhodospirillales	2.1	1.9	2.8	2.6	3.0	2.6	2.5	1.3
Gammaproteobacteria, Alteromonadales	4.9	4.6	1.2	0.5	0.4	0.3	2.0	-15.6
SAR406, ZA3648c	1.0	1.1	2.1	2.0	2.3	2.4	1.8	2.3
Chloroflexi, Dehalococcoidales	1.0	1.0	1.5	1.4	2.1	1.8	1.5	1.7
Euryarchaeota, Thermoplasmata, E2	0.6	1.1	1.1	1.1	1.9	2.8	1.4	4.4
Crenarchaeota, MBGA	1.2	1.0	1.7	1.4	1.7	1.5	1.4	1.3
Gammaproteobacteria, Thiohalorhabdales	0.9	1.2	1.2	1.0	1.4	2.4	1.4	2.8
Alphaproteobacteria, Kiloniellales	3.3	4.0	0.2	0.1	0.0	0.0	1.3	-93.6
Gammaproteobacteria, Thiotrichales	0.7	0.8	1.3	1.1	1.3	1.5	1.1	2.0
Thaumarchaeota, Cenarchaeales	1.0	0.5	1.3	1.3	1.3	1.2	1.1	1.2
Deltaproteobacteria, Desulfobacterales	0.9	1.1	1.4	1.1	1.1	1.0	1.1	1.2
Alphaproteobacteria, Rhodobacterales	4.1	1.4	0.3	0.1	0.1	0.0	1.0	-85.7
Bacteroidetes, Flavobacteriales	0.2	2.8	0.4	0.4	0.3	0.9	0.8	3.7
Planctomycetes, Planctomycetales	0.0	4.5	0.0	0.1	0.0	0.0	0.8	-11.4
Deltaproteobacteria, Entotheonellales	0.6	0.6	1.0	0.7	0.9	0.8	0.8	1.2
Acidobacteria, iii1-15	0.6	0.4	0.7	0.6	0.8	0.7	0.6	1.2
Betaproteobacteria, Methylophilales	0.0	3.8	0.0	0.0	0.0	0.0	0.6	-1.2
Chloroflexi, H39	0.2	0.3	0.5	0.6	0.6	0.7	0.5	3.5

Table S4 continued

Prefilter fraction (>1.6 μm)

Taxon	Filtered water volume*						Average (all)	Fold Change
	0.05L	0.1L	0.5L	1L	2L	5L		
Gammaproteobacteria, Vibrionales	62.2	49.6	38.5	4.7	2.6	1.9	26.6	-33.4
Gammaproteobacteria, Chromatiales	2.2	1.4	2.8	27.9	58.1	38.7	21.8	17.6
Gammaproteobacteria, Alteromonadales	8.1	8.8	7.5	2.3	1.6	1.8	5.0	-4.5
SAR406, Arctic96B-7	0.9	4.2	7.0	7.0	4.2	6.6	5.0	7.7
Gammaproteobacteria, Oceanospirillales	7.5	7.9	4.4	3.4	2.0	1.7	4.5	-4.4
Euryarchaeota, Thermoplasmata, E2	0.4	2.0	4.5	6.2	4.2	7.1	4.1	19.5
Unassigned	0.6	2.6	5.3	5.9	3.3	4.4	3.7	7.1
Gammaproteobacteria, Thiohalorhabdales	0.6	1.4	3.3	7.5	2.6	4.6	3.3	7.6
Firmicutes, Bacillales	5.1	4.3	1.0	0.3	0.0	0.5	1.9	-10.2
Epsilonproteobacteria, Campylobacterales	0.1	0.2	0.9	2.7	4.2	1.9	1.7	20.0
Alphaproteobacteria, Rickettsiales	0.4	1.1	2.5	2.3	1.4	2.0	1.6	4.7
Bacteroidetes, Flavobacteriales	0.3	1.0	1.5	3.0	1.5	1.4	1.5	4.2
Planctomycetes, Brocadiales	0.6	1.6	2.4	1.6	1.2	1.3	1.4	2.3
Gammaproteobacteria, Thiotrichales	0.0	0.5	1.3	2.4	1.4	2.5	1.3	65.0
Alphaproteobacteria, Rhodobacterales	3.2	2.4	0.7	0.1	0.2	0.1	1.1	-49.0
Alphaproteobacteria, Rhodospirillales	0.3	0.8	1.5	1.9	0.6	1.2	1.0	4.5
SAR406, ZA3648c	0.2	0.6	1.3	2.4	0.6	0.9	1.0	5.2
Chloroflexi, SAR202,	0.2	0.7	1.6	1.3	0.5	0.9	0.9	4.1
Deltaproteobacteria, Myxococcales	0.2	0.4	0.5	1.4	0.5	1.4	0.8	8.5
Planctomycetes, Phycisphaerales	0.2	0.5	0.4	1.9	0.2	1.1	0.7	6.3
Actinobacteria, Acidimicrobiales	0.2	0.3	1.2	0.8	0.7	0.8	0.7	4.8
Alphaproteobacteria, Kiloniellales	1.6	1.3	0.3	0.0	0.0	0.1	0.6	-23.2
Alphaproteobacteria, Sphingomonadales	1.0	0.7	0.0	0.0	0.0	1.2	0.5	1.2
Bacteroidetes, Bacteroidales	0.1	0.1	0.4	0.4	0.8	0.9	0.5	10.9
